# Supplementary material for: The effect of supply chain risks management practices on operational performance of pharmaceutical manufacturing companies in Addis Ababa, Ethiopia: Analytical cross-sectional study
Source: PLoS One. 2025 May 8;20(5):e0321311. doi: 10.1371/journal.pone.0321311 (PMC12061155; doi:10.1371/journal.pone.0321311)
Supplement: S1 Table — (ZIP) [file pone.0321311.s001.zip › Supplementary file figure 1.pdf]

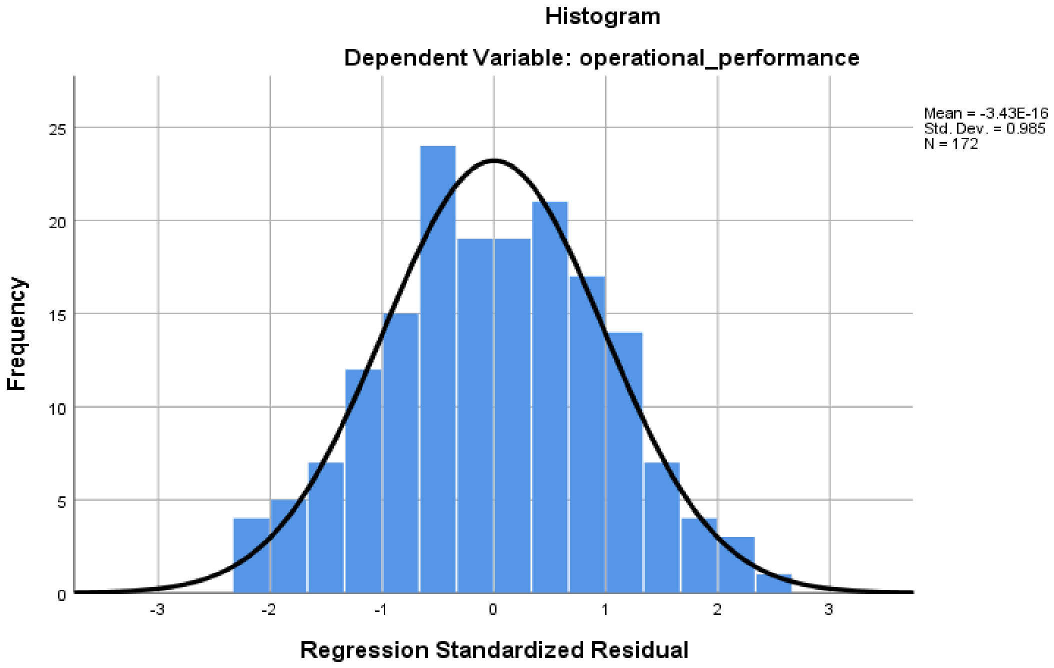

Supplementary file figure 1: Histogram on a normal distribution of residuals against the predicted dependent variable scores in pharmaceutical companies of Addis Ababa, Ethiopia, 2023(N=172)
