# Supplementary material for: The effect of supply chain risks management practices on operational performance of pharmaceutical manufacturing companies in Addis Ababa, Ethiopia: Analytical cross-sectional study
Source: PLoS One. 2025 May 8;20(5):e0321311. doi: 10.1371/journal.pone.0321311 (PMC12061155; doi:10.1371/journal.pone.0321311)
Supplement: S1 Table — (ZIP) [file pone.0321311.s001.zip › Supplementary file survey questioner annex.pdf]

**Supplementary file survey questioner annex:** Questioner used to collect quantative and qualitative data from pharmaceutical companies of Addis Ababa, Ethiopia, 2023 (N=172)

**Section I- Demographic data**

**Instructions:** Please answer questions by putting a tick [√] in the appropriate box or by writing in the space provided

1. Gender: Male Female
2. Age _________
3. Educational Qualification: Diploma____ degree_____ master and above_______
4. Year of service in your section ___________
5. In which section of the supply chain department do you currently working in?________

**Instructions**: Please read each statement carefully and show the extent of your agreement on the statements by putting a tick mark (√) in the boxes of your choice from listed out rating scales.

**Section II** - Rate the extent that your company experienced/exposed the following supply chain related risks. **Key**: (1-Strongly Disagree 2-Disagree 3-Neutral 4-Agree 5-Strongly Agree)

| | **Supply Chain Related Risk** | 1 | 2 | 3 | 4 | 5 |
|---|---|---|---|---|---|---|
| | **Demand side risks/DR** | | | | | |
| DR-1 | Customer's unanticipated demand for the company product | | | | | |
| DR-2 | There are insufficient or distorted information in company | | | | | |
| DR-3 | Company forecasting risks (Lead times, product variety, short life cycles, information distortion, exaggeration of demand) | | | | | |
| | **Supply Side Risks/SR/** | | | | | |
| SR-1 | Deprived logistics performance of suppliers for company | | | | | |
| SR-2 | Company dependency on key suppliers only | | | | | |
| SR-3 | There are poor communication of company with the suppliers | | | | | |
| SR-4 | Company lack partnership/coordination with suppliers | | | | | |
| SR-5 | Company suppliers have products quality problems | | | | | |
| SR-6 | Company suppliers increases/ rating high products prices | | | | | |
| SR-7 | Raw Products availability fluctuations for the company | | | | | |
| | **Regulatory and legal related supply chain risks** | | | | | |

| | | | | | | |
|---|---|---|---|---|---|---|
| RLBR-1 | Company committing government administrative barriers to the setup and fulfill the customer satisfaction | | | | | |
| RLBR-2 | Regulatory system changes due to the-introduction of new laws | | | | | |
| | **Financial-related supply chain risks** | | | | | |
| F-1 | Company faces the dynamic foreign exchange rates | | | | | |
| F-2 | Company faces the bank's interest rate fluctuation | | | | | |
| F-3 | Company committing financial restriction to avail products | | | | | |
| F-4 | Company is facing high in freight charges of products | | | | | |
| | **Infrastructure related supply chain risks** | | | | | |
| IR-1 | Breakdown of external IT infrastructure | | | | | |
| IR-2 | Breakdown of internal IT infrastructure- | | | | | |
| IR-3 | Downtime or loss of own production capacity due to local disruptions (e.g., fire, strike) | | | | | |
| IR-4 | Loss of own production capacity due to technical reasons | | | | | |
| IR-5 | Lack of proper storage area with adequate facilities | | | | | |
| IR-6 | Infrastructure unavailability(water , electricity IT, vehicle, road, Equipment) | | | | | |
| IR-7 | Lack of information transparency between logistics and marketing | | | | | |
| IR-8 | Paperwork and scheduling | | | | | |
| | **Catastrophic risks/CR** | | | | | |
| CR-1 | Political instability, war, civil unrest or other sociopolitical Crises. | | | | | |
| CR-2 | Diseases or epidemics | | | | | |
| CR-3 | Natural disasters | | | | | |
| | **Production risk/PR** | | | | | |
| PR-1 | Company working machine breakdown | | | | | |
| PR-2 | Outsourcing key business processes (delay, risk of intellectual property) | | | | | |
| PR-3 | Insufficient maintenance of working equipment's | | | | | |
| PR-4 | Lack of skilled workers | | | | | |
| PR-5 | Low Production capabilities/capacity | | | | | |

**Section III-Supply chain Risk mitigation practices**

**Key**: (1-Strongly Disagree 2-Disagree 3-Neutral 4-Agree 5-Strongly Agree)

| **Supply chain risks management practices** | **Level of agreement** | | | | |
|---|---|---|---|---|---|
| | **1** | **2** | **3** | **4** | **5** |
| The company treasures have a collaborative relationship with its key suppliers | | | | | |
| The company collaborate with its key suppliers in the areas of sharing risks | | | | | |
| There is considerable trust between the company and its key suppliers | | | | | |
| The supply chain risks for the organization are known and documented | | | | | |
| In the company, risk management practices are inclusive and participatory | | | | | |
| The company categorize the supply chain risks as high, medium & low | | | | | |
| Risk awareness practices are matured or common in the organization | | | | | |
| The company maintains buffer stocks for both raw and finished items | | | | | |
| In company, inventory is only maintained for long-lead time & critical items | | | | | |
| The buffer stocks are maintained considering to minimizing stock holding cost, obsceneness and damage in the company | | | | | |
| The company identifies the potential supplier risks reports during vendor appraisals | | | | | |
| The company undertakes continuous supply chain performance audits (quality, cost, delivery) | | | | | |
| The company maintains a backup supplier for pharmaceutical products | | | | | |
| The supply chain contingency planning is a critical of for the company | | | | | |
| The company plan minimize loss, safe assets and mitigate risks | | | | | |
| Company considers insurance as a key for mitigating supply chain risks | | | | | |

**Section IV- Pharmaceutical manufacturing's Performance**

Key: (1-Strongly Disagree 2-Disagree 3-Neutral 4-Agree 5-Strongly Agree)

| Operational performance of organizations | Level of agreement | | | | |
|---|---|---|---|---|---|
| | SD | DA | N | A | SA |
| The company avail the products with the reasonable price | | | | | |
| The company have strategies to minimize the cost of operations, invent and warehouse | | | | | |
| The company ensure product quality at each stage | | | | | |
| The company provides defect free products for customers | | | | | |
| Company identified key processes and activities that influence produc quality | | | | | |
| The company produce multiple variant products | | | | | |
| The company undertake proactive and reactive adaptation of settings to deal with uncertainties | | | | | |
| Company is flexible to accommodate the changes in the working Environment | | | | | |
| The company utilize outsourcing for non-competence activities | | | | | |
| Company Properly execute the clients' order | | | | | |
| Company satisfies the client's demands for products | | | | | |

**Part 2. Questionnaire for the qualitative study**

Interviewed by principal investigator Mr. ____________________________

1. How do you describe supply chain risks of pharmaceutical manufacturing companies?
2. What are the major possible supply chain risks for your companies
3. Lists the supply risks you committed and describes them for us how they faced your organization?
4. How do you identify and monitor supply chain risks in your organizations?
5. What risks mitigation practices did you implement in your manufacturing companies?
6. What are opportunities and challenges in supply chain risks mitigation practices for your organizations
7. What is your recommendation for pharmaceutical manufacturers companies for effective supply chain risks mitigation practices?

**Thank you!**
