# Supplementary material for: The effect of supply chain risks management practices on operational performance of pharmaceutical manufacturing companies in Addis Ababa, Ethiopia: Analytical cross-sectional study
Source: PLoS One. 2025 May 8;20(5):e0321311. doi: 10.1371/journal.pone.0321311 (PMC12061155; doi:10.1371/journal.pone.0321311)
Supplement: S1 Table — (ZIP) [file pone.0321311.s001.zip › Supplementary file Table 1.pdf]

Supplementary file Table 1: Supply chain risks in pharmaceutical companies of Addis Ababa, Ethiopia, 2023 (N=172)

Supply chain risks	Level of agreement					
	**SD (%)**	**DA (%)**	**N (%)**	**A (%)**	**SA (%)**	**Mean**
**Demand side supply chain risks**						
Customer's unanticipated or very volatile demand for the company product	4 (2.3)	32(18.6)	25 (14.5)	84 (48.8)	27(15.7)	3.57
There are insufficient or distorted information in company	7(4.1)	35(20.3)	15(8.7)	79(45.9)	36(20.9)	3.59
Company forecasting risks (Lead times, product variety, short life cycles, information distortion, exaggeration of demand)	4(2.3)	19(11.0)	29(16.9)	87(50.6)	33(19.2)	3.73
**Supply Side supply chain risks**						
Deprived logistics performance of suppliers for company	4(2.3)	19(11.0)	29(16.9)	87(50.6)	33(19.2)	3.73
Company dependency on key suppliers only	4(2.3)	38(22.1)	29(16.9)	82(47.7)	19(11.0)	3.43
There are poor communication of company with the suppliers	8(10.5)	43(25.0)	44(25.6)	56(32.6)	11(6.4)	2.99
Company lack partnership/coordination with suppliers	11(6.4)	7(33.1)	29(16.9)	53(30.8)	22(12.8)	3.10
Company suppliers have products quality problems	11(6.4)	57(33.1)	20(11.6)	64(37.2)	20(11.6)	3.15
Company suppliers increases/ rating high products prices	7(4.1)	20(11.6)	42(24.4)	76(44.2)	27(15.7)	3.56
Raw Products availability fluctuations for the company	6(3.5)	38(22.1	20(11.6)	79(45.9)	29(16.9)	3.51
**Regulatory and legal related supply chain risks**						
Company committing government administrative barriers to the setup and fulfil the customer satisfaction	-	35(20.3)	45(26.2)	74(43.0)	18(10.5)	3.44

Regulatory system changes due to the-introduction of new laws	1(0.6)	21(12.2)	46(26.7)	75(43.6)	29(16.9	3.64
**Financial-related supply chain risks**						
Company faces the dynamic foreign exchange rates	2(1.2)	56(32.6)	31(18.0)	63(36.6)	20(11.6)	3.25
Company faces the bank's interest rate fluctuation	6(3.5)	64(37.2)	38(22.1)	45(26.2)	19(11.0)	3.04
Company committing financial restriction to avail products	3(1.7)	54(31.4)	36(20.9)	56(32.6)	23(13.4)	3.24
Company is facing high in freight charges of products	4(2.3)	34(19.8)	32(18.6)	80(46.5)	22(12.8)	3.48
**Infrastructure related supply chain risks**						
Breakdown of external IT infrastructure	16(9.3)	44(25.6)	47(27.3)	50(29.1)	15(8.7)	3.02
Breakdown of internal IT infrastructure-	22(2.8)	32(18.6)	40(23.3)	65(37.8)	13(7.6)	3.09
Downtime or loss of own production capacity due to local disruptions (e.g., fire, strike)	25(14.5)	45(26.2)	25(14.5)	53(30.8)	24(14.0)	3.03
Loss of own production capacity due to technical reasons	21(12.2)	35(20.3)	55(32.0)	45(26.2)	16(9)	3.00
Lack of proper storage area with adequate facilities	39(22.7)	35(20.3)	43(25.0)	38(22.1)	17(9.9)	2.76
Infrastructure unavailability(water , electricity IT, vehicle, road, Equipment)	21(12.2)	36(20.9)	37(21.5)	57(33.1)	21(12.2)	3.12
Lack of information transparency between logistics and marketi	15(8.7)	58(33.7)	59(34.3)	35(20.3)	5(2.9)	2.75
Paperwork and scheduling	24(14.0)	54(31.4)	46(26.7)	34(19.8)	14(8.1)	2.77
**Catastrophic side supply chain risks**						
Political instability, war, civil unrest or other sociopolitical Crise	2(1.2)	15(8.7)	21(12.2)	76(44.19)	58(33.7)	4.01
Diseases or epidemics	28(16.3)	66(38.4)	22(12.8)	42(24.4)	14(8.1)	2.70

Natural disasters	63(36.6)	75(43.6)	15(8.7)	15(8.7)	4(2.3)	1.97
**Production side supply chain risks**						
Company working machine breakdown	1(0.6)	54(31.4)	36(20.9)	64(37.2)	17(9.9)	3.24
Outsourcing key business processes (delay, risk of intellectual property)	25(14.5)	58(33.7)	43(25.0)	44(25.6)	2(1.2)	2.65
Insufficient maintenance of working equipment’s	7(4.1)	51(29.7)	23(13.4)	73(42.4)	18(10.5)	3.26
lack of skilled workers	20(11.6)	76(44.2)	23(13.4)	47(27.3)	6(3.5)	2.67
Low Production capabilities/capacity	16(9.3)	89(51.7)	24(14.0)	37(21.5)	6(3.5)	2.58
