# Supplementary material for: The effect of supply chain risks management practices on operational performance of pharmaceutical manufacturing companies in Addis Ababa, Ethiopia: Analytical cross-sectional study
Source: PLoS One. 2025 May 8;20(5):e0321311. doi: 10.1371/journal.pone.0321311 (PMC12061155; doi:10.1371/journal.pone.0321311)
Supplement: S1 Table — (ZIP) [file pone.0321311.s001.zip › Supplementary file Table 2.pdf]

Supplementary file Table 2: Supply chain Risk mitigation practices in pharmaceutical firms of Addis Ababa, Ethiopia, 2023(N=172)

Supply chain risks management practices	Level of agreement				
	SD (%)	DA (%)	N (%)	A (%)	SA (%)
The company treasures have a collaborative relationship with its key suppliers	2(1.2)	34(19.8)	52(30.2)	73(42.4)	11(6.4)
The company collaborate with its key suppliers in the areas of sharing risks	2(1.2)	22(12.8)	47(27.3)	87(50.6)	14(8.1)
There is considerable trust between the company and its key suppliers	10(5.8)	35(20.3)	20(11.6)	86(50.0)	21(12.2)
The supply chain risks for the organization are known and documented	5(2.9)	74(43.0)	30(17.4)	49(28.5)	14(8.1)
In the company, risk management practices are inclusive and participatory	5(2.9)	77(44.81)	38(22.1)	45(26.2)	7(4.1)
The company categorize the supply chain risks as high, medium & low	4(2.3)	62(36.0)	49(28.5)	47(27.3)	10(5.8)
Risk awareness practices are matured or common in the organization	8(4.7)	65(37.8)	58(33.7)	35(20.3)	6(3.5)
The company maintains buffer stocks for both raw and finished items	9(5.2)	7(4.1)	48(27.9)	50(29.1)	7(4.1)
In company, inventory is only maintained for long-lead time & critical items	4(2.3)	69(40.1)	45(26.2)	46(26.7)	8(4.7)
The buffer stocks are maintained considering to minimizing stock holding cost, obsceneness and damage in the company	5(2.9)	59(34.3)	47(27.3)	53(30.8)	8(4.7)
The company identifies the potential supplier risks reports during vendor appraisals	5(2.9)	51(29.7)	44(25.6)	63(36.6)	9(5.2)
The company undertakes continuous supply chain performance audits (quality, cost, delivery)	16(9.3)	87(50.6)	27(15.7)	37(21.5)	5(2.9)
The company maintains a backup supplier for pharmaceutical products	8(4.7)	47(27.3)	51(29.7)	62(36.0)	4(2.3)
The supply chain contingency planning is a critical of for the company	5(2.9)	45(26.2)	43(25.0))	75(43.6)	4(2.3)
The company plan minimize loss, safe assets and mitigate risks	5(2.9)	60(34.9)	39(22.7)	59(34.3)	9(5.2)
Company considers insurance as a key for mitigating supply chain risks	7(4.1)	37(21.5)	32(18.6)	92(53.5)	4(2.3)
