# Supplementary material for: The effect of supply chain risks management practices on operational performance of pharmaceutical manufacturing companies in Addis Ababa, Ethiopia: Analytical cross-sectional study
Source: PLoS One. 2025 May 8;20(5):e0321311. doi: 10.1371/journal.pone.0321311 (PMC12061155; doi:10.1371/journal.pone.0321311)
Supplement: S1 Table — (ZIP) [file pone.0321311.s001.zip › Supplementary file Table 3.pdf]

Supplementary file Table 3: The result of multicollinearity test for independent variables in pharmaceutical companies of Addis Ababa, Ethiopia, 2023 (n=172)

Variables	Collinearity Statistics Tolerance	VIF
Demand risks	.762	1.312
Supply risks	.605	1.653
Regulatory risks	.825	1.212
Infrastructure risks	.506	1.978
Catastrophic risks	.762	1.313
Production risks	.616	1.622
Financial risks	.750	1.333
