# Supplementary material for: The effect of supply chain risks management practices on operational performance of pharmaceutical manufacturing companies in Addis Ababa, Ethiopia: Analytical cross-sectional study
Source: PLoS One. 2025 May 8;20(5):e0321311. doi: 10.1371/journal.pone.0321311 (PMC12061155; doi:10.1371/journal.pone.0321311)
Supplement: S1 Table — (ZIP) [file pone.0321311.s001.zip › Supplementary file Table 4.pdf]

Supplementary file Table 4: Bivariate correlation among the independent and dependent variable in pharmaceutical companies of Addis Ababa, Ethiopia, 2023 (n=172)

	DR	SR	RR	IR	CR	PR	FR
DR	1						
SR	.414**	1	.				
RR	095	.134	1				
IR	. 163*	.468**	. 076	1			
CR	-.038	-.117	-.103	.203**	1		
PR	.001	.299**	.170*	.572*	.090	1	
FR	-.067	-.050.	296**	-.091	275 *	.080	1
OP	-.304**	-.187**	.086	-.494**	.224**	. 211*	-.485*

Notice: DR=Demand risk, SR=Supply risk, RR= Regulatory risk, IR=Infrastructure risk, CR=Catastrophic risk, PR= Production risk, FR= Financial risk & OP= operational performance & **Correlation is significant at the 0.01 level (2-tailed). *Correlation is significant at the 0.05 level (2-tailed).
