# Supplementary material for: The effect of supply chain risks management practices on operational performance of pharmaceutical manufacturing companies in Addis Ababa, Ethiopia: Analytical cross-sectional study
Source: PLoS One. 2025 May 8;20(5):e0321311. doi: 10.1371/journal.pone.0321311 (PMC12061155; doi:10.1371/journal.pone.0321311)
Supplement: S1 Table — (ZIP) [file pone.0321311.s001.zip › Supplementary file Table 5.pdf]

Supplementary file Table 5: Significance level for multiple correlation coefficient-ANOVA in pharmaceutical companies of Addis Ababa, Ethiopia, 2023 (N=172)

Model	Sum of Square	Df	Mean Square	F	Sig.
Regression	15.654	7	2.236	9.935	.000b
Residual	36.915	164	.225		
Total	52.569	171			

a Predictors: (Constant), Demand, Supply, Regulatory, Infrastructure, Catastrophic, Production, Financial risks
