# Supplementary material for: The effect of supply chain risks management practices on operational performance of pharmaceutical manufacturing companies in Addis Ababa, Ethiopia: Analytical cross-sectional study
Source: PLoS One. 2025 May 8;20(5):e0321311. doi: 10.1371/journal.pone.0321311 (PMC12061155; doi:10.1371/journal.pone.0321311)
Supplement: Attachment — Submitted filename: Response to reviewer and Editor.docx [file pone.0321311.s011.docx]

**Esteemed editor and reviewers, we extend our sincere gratitude for your invaluable and constructive suggestions and comments. Your input has been carefully considered, and we have made effort to address your concerns as per your guidance, incorporating them into the rebuttal letter and the manuscript documents.**

**Raised comments suggestion and the proposed response**

1. Please ensure that your manuscript meets PLOS ONE's style requirements, including those for file naming.

**Response:** *Thank you for reminding us and for your suggestion. We have carefully reviewed the writing style you mentioned and have made adjustments to align with it.*

2. PLOS requires an ORCID iD for the corresponding author in Editorial Manager on papers submitted after December 6th, 2016. Please ensure that you have an ORCID iD and that it is validated in Editorial Manager. To do this, go to ‘Update my Information’ (in the upper left-hand corner of the main menu), and click on the Fetch/Validate link next to the ORCID field. This will take you to the ORCID site and allow you to create a new iD or authenticate a pre-existing iD in Editorial Manager.

**Response:** *We have verified and authenticated the corresponding author ORCID in the PLOS ONE. ORCID:* [*https://orcid.org/0000-0002-9218-9669*](https://orcid.org/0000-0002-9218-9669)

3. Thank you for stating the following in your Competing Interests section:  The authors declare that they have no known competing financial interests or personal relationships that could have appeared to influence the work reported in this paper. Please complete your Competing Interests on the online submission form to state any Competing Interests. If you have no competing interests, please state ""The authors have declared that no competing interests exist."", as detailed online in our guide for authors at <http://journals.plos.org/plosone/s/submit-now>
    This information should be included in your cover letter; we will change the online submission form on your behalf.

**Response:** *We have addressed any potential competing interests. The statement "The authors have declared that no competing interests exist" was submitted through manuscript document and cover latter pre your respond to my Gmail request response “Thank you for reaching out.
 If the competing interest statement is already included within your paper you may now resubmit (as it is). I will gladly updated the online submission on your behalf*”

4. Please amend the title either on the online submission form or in your so that they are identical

**Response:** *Thank you for reminding us to verify the consistency between the online submission and the manuscript document. We have reviewed and corrected it as per your comments.*

5. We note that you have indicated that there are restrictions to data sharing for this study. For studies involving human research participant data or other sensitive data, we encourage authors to share de-identified or anonymized data. However, when data cannot be publicly shared for ethical reasons, we allow authors to make their data sets available upon request. Before we proceed with your manuscript, please address the following prompts:

a) If there are ethical or legal restrictions on sharing a de-identified data set, please explain them in detail (e.g., data contain potentially identifying or sensitive patient information, data are owned by a third-party organization, etc.) and who has imposed them (e.g., a Research Ethics Committee or Institutional Review Board, etc.). Please also provide contact information for a data access committee, ethics committee, or other institutional body to which data requests may be sent.

**Response:** *All relevant data are within the manuscript and its Supporting Information files. The data is accessible from the authors and can be shared with no ethical or legal restrictions upon request. The data were collected and handled by the authors. So, it is not owned by a third-party organization.*

b) If there are no restrictions, please upload the minimal anonymized data set necessary to replicate your study findings to a stable, public repository and provide us with the relevant URLs, DOIs, or accession numbers. Please see <http://www.bmj.com/content/340/bmj.c181.long> for guidelines on how to de-identify and prepare clinical data for publication. For a list of recommended repositories, please see <https://journals.plos.org/plosone/s/recommended-repositories>. You also have the option of uploading the data as Supporting Information files, but we would recommend depositing data directly to a data repository if possible. Please update your Data Availability statement in the submission form accordingly.

**Response:** *We have revised our statement on data availability and sharing in both the submission platform and the manuscript document per your recommendation.*

6. Please include captions for your Supporting Information files at the end of your manuscript, and update any in-text citations to match accordingly.

**Response:** *Thank you for your suggestion. We have incorporated the captions for the supporting information files at the end of the manuscript document as per your recommendation*.

7. Please review your reference list to ensure that it is complete and correct. If you have cited papers that have been retracted, please include the rationale for doing so in the manuscript text, or remove these references and replace them with relevant current references. Any changes to the reference list should be mentioned in the rebuttal letter that accompanies your revised manuscript. If you need to cite a retracted article, indicate the article’s retracted status in the References list and also include a citation and full reference for the retraction notice.

**Response:** *Thank you for your suggestions. We have thoroughly reviewed all references and did not come across the retracted reference.*
**Reviewers' comments:**
Reviewer's Responses to Questions

**Comments to the Author**
1. Is the manuscript technically sound, and do the data support the conclusions?
The manuscript must describe a technically sound piece of scientific research with data that supports the conclusions. Experiments must have been conducted rigorously, with appropriate controls, replication, and sample sizes. The conclusions must be drawn appropriately based on the data presented.

Reviewer #1: Yes

Reviewer #2: Yes

**Response**: *Thank you for providing your review. I sincerely appreciate your understanding and encouragement.*

2. Has the statistical analysis been performed appropriately and rigorously?

Reviewer #1: Yes

Reviewer #2: Yes

**Response: *Thanks***

3. Have the authors made all data underlying the findings in their manuscript fully available?

The [PLOS Data policy](http://track.editorialmanager.com/CL0/http:%2F%2Fwww.plosone.org%2Fstatic%2Fpolicies.action%23sharing/1/010f0194ad58a13f-371aa8fc-8c09-49a6-b574-0ab128bb131f-000000/vWEKDvmmpplgiRV9tDUEGWBnqFHjq-EFKJ5MvdJORGk=195) requires authors to make all data underlying the findings described in their manuscript fully available without restriction, with rare exception (please refer to the Data Availability Statement in the manuscript PDF file). The data should be provided as part of the manuscript or its supporting information, or deposited to a public repository. For example, in addition to summary statistics, the data points behind means, medians and variance measures should be available. If there are restrictions on publicly sharing data—e.g. participant privacy or use of data from a third party—those must be specified.

Reviewer #1: Yes

Reviewer #2: Yes

**Response**: *Thanks*

4. Is the manuscript presented in an intelligible fashion and written in standard English?

PLOS ONE does not copyedit accepted manuscripts, so the language in submitted articles must be clear, correct, and unambiguous. Any typographical or grammatical errors should be corrected at revision, so please note any specific errors here.

Reviewer #1: Yes

Reviewer #2: Yes

**Response**: Thanks

5. Review Comments to the Author

**Reviewer #1:**

1. Interesting work and findings as it provides insight of supply chain risk management in a geographical context that is less explored. Abstract is clear summarizing the work but can be improved with highlights on how these findings contribute to global supply chain risk management field and policy/industry practices.
   **Response**: *Thank you for your suggestion. We have integrated your concern into the abstract, conclusion section, and emphasized it.*
2. For literature review, please conduct comparison and critically analyse the similar studies in other countries/industries to provide more context and shows the needs of this research. **Response:** *Thank you for your input. We have endeavored to incorporate your recommendation by reviewing study findings from various countries or industries.*
3. Methods are clear but it should explain more on limitations acknowledged in the paper and how this impacted the findings.

**Response**: *We have detailed the limitations of the study and discussed how they impact the results of our research.*

4. The findings of this study should be relate or associated with current or existing supply chain risk management theories or model in order to show the significant of the results to the SCRM field. Discussion on how the findings could be relevant to other developing countries or even to global supply chain facing similar risks. From the findings of this risk, authors can discuss potential application of technology such as AI to mitigate the risks.

**Response**: *Thank you for your valuable input and suggestions, particularly regarding AI. We have made an effort to address your points by incorporating your suggestions into the main document.* *Dear reviewer, following our research, we have explored studies conducted in developing countries. In light of limitations observed in previous studies within this domain, we found and included developing nations like Ghana and Ethiopia. Additionally, we have considered the study of the electricity industry supply chain in Kenya; however, it differed from the pharmaceutical products supply chain. Further we have enhanced our findings by leveraging insights from developed and middle-income countries.*

**Reviewer #2**:

5. An article of high substantive level and with an appropriate literature review. Alternatively, one could consider a more readable presentation of the issue of assessing the reliability of the research tool (survey questionnaire) in the form of a table. One could also consider the justification for using so many statistical methods in verifying research hypotheses. I propose to indicate more clearly the direction of future research in the undertaken research area.

**Response**: *Thank you for your feedback and suggestions. The survey questionnaire used has been included in the supplementary file for reference. A potential future research recommendation was written under the section titled "Research Strengths and Limitations."*

**Thank you immensely for your valuable input!**
